# Engineered T Cell Therapy for Cancer in the Clinic

**DOI:** 10.3389/fimmu.2019.02250

**Published:** 2019-10-11

**Authors:** Lijun Zhao, Yu J. Cao

**Affiliations:** State Key Laboratory of Chemical Oncogenomics, Key Laboratory of Chemical Genomics, Peking University Shenzhen Graduate School, Shenzhen, China

**Keywords:** engineered T cells, CAR-T cell, TCR-T cell, clinic, immunotherapy

## Abstract

T cells play a key role in cell-mediated immunity, and strategies to genetically modify T cells, including chimeric antigen receptor (CAR) T cell therapy and T cell receptor (TCR) T cell therapy, have achieved substantial advances in the treatment of malignant tumors. In clinical trials, CAR-T cell and TCR-T cell therapies have produced encouraging clinical outcomes, thereby demonstrating their therapeutic potential in mitigating tumor development. This article summarizes the current applications of CAR-T cell and TCR-T cell therapies in clinical trials worldwide. It is predicted that genetically engineered T cell immunotherapies will become safe, well-tolerated, and effective therapeutics and bring hope to cancer patients.

## Introduction

The microenvironment of solid tumors contains an abundant fibrous matrix and immunosuppressive cells, which can protect the tumor tissue and resist immune cell attack (1). For example, in the pancreatic cancer microenvironment, the content of hyaluronan (HA) increases significantly, which impedes the infiltration of the immune cells (2). Immune checkpoint receptors on tumor cells or immunosuppressive cells can inhibit T cells, by binding to negative regulatory ligands on T cells (3). In this low-oxygen, acidic tumor microenvironment (TME) that lack essential amino acids, infiltrating T cells experience anergy, exhaustion, senescence, and stemness, making it challenging to achieve the desired tumor killing effect (4). There is still no cure for these diseases, and treatments only control their malignant development in various ways. Traditional methods of cancer treatment include surgical resection, radiotherapy and chemotherapy, small molecule targeted drugs, monoclonal antibodies, and hematopoietic stem cell transplantation. Surgical resection is effective for early-stage patients but not for metastatic cancer cells; radiotherapy and chemotherapy are more common but have poor selectivity and produce substantial damage to normal tissues, and targeted drugs (including small molecules and monoclonal antibodies) have better comprehensive efficacy and fewer toxic side effects but can also encourage gene mutation of tumor cells, drug tolerance, and so on (5). Hematopoietic stem cell transplantation is effective; however, choosing a donor is often difficult, and the graft is prone to rejection after the operation. Cellular immunotherapy is at the forefront of cancer therapy (6).

In recent years, great progress has been made in cancer treatment. Researchers not only have a better understanding of various solid and blood cancers but also have expanded the scope and impact of various immunotherapy strategies (7). Immunotherapy is the fastest and most remarkable developing field. Immunotherapy has generated new therapeutic options for a series of solid tumor and blood cancer patients; as such, the 2018 Nobel Prize in Physiology or Medicine was awarded to James P. Allison and Tasuku Honjo, who discovered how to harness the body's immune system to fight cancer, highlighting the importance of research progress in the field of immunotherapy (8).

Among immunotherapies, the programmed cell death protein 1 (PD-1) and programmed cell death ligand 1 (PD-L1) checkpoint inhibitors have been shown to be the primary treatment for advanced lung cancer (9–11). Immunotherapy combined with antibody therapy targeting PD-1 or PD-L1 (ClinicalTrials.gov number: NCT02366143 and NCT02578680) significantly increased median progression-free survival and 1-year overall survival (9–11). On September 30, 2018, the U.S. Food and Drug Administration (FDA) approved a new anti-PD-1 checkpoint inhibitor (Cemiplimab; Libtayo) for skin squamous cell carcinoma (Table 1).

**Table 1:** The FDA has approved a variety of new immunotherapies.

**Trade name**	**Generic name**	**Target**	**Disease**	**Approved by FDA**
Provenge	Sipuleucel-T	PAP	Asymptomatic or minimally symptomatic metastatic castrate resistant (hormone refractory) prostate cancer	2010.4
Yervoy	Ipilimumab	CTLA-4	Adult patients with inoperable or metastatic melanoma	2011.3
			Pediatric patients aged 12 years and older with unresectable or metastatic melanoma	2017.7
Keytruda	Pembrolizumab	PD-1	Advanced or unresectable melanoma	2014.9
			Classic Hodgkin's lymphoma	2017.3
			Recurrent or metastatic cervical cancer	2018.6
Blincyto	Blinatumomab	CD19, CD3	B-cell precursor ALL	2014.1
			MRD positive B cell precursor ALL	2018.3
Opdivo	Nivolumab	PD-1	Advance melanoma	2014.1
			NSCLC	2015.3
			Colorectal cancer	2017.8
Tecentriq	Atezolizumab	PD-L1	NSCLC	2018.1
			First-line treatment for extensive stage small cell lung cancer	2019.3
Bavencio	Avelumab	PD-L1	Metastatic MCC	2017.3
			Locally advanced or metastatic urothelial carcinoma	2017.5
Imfinzi	Durvalumab	PD-L1	Locally advanced or metastatic bladder carcinoma	2017.5
			Stage 3 non-small cell lung cancer that is stable after surgery, chemotherapy, or radiation	2018.2
Kymriah	Tisagenlecleucel	CD19	B-cell precursor ALL	2017.8
Yescarta	Axicabtagene Ciloleucel	CD19	Adults with relapsed or refractory large B-cell lymphoma	2017.1
Opdivo & Yervoy	Nivolumab & Ipilimumab	PD-1, CTLA-4	Intermediate and poor-risk advanced renal cell carcinoma	2018.4
Keytruda & Inlyta	Pembrolizumab & Axitinib	PD-1, VEGFR	Advanced RCC	2019.4

*PAP, prostate acid phosphatase; CTLA-4, cytotoxic T-lymphocyte-associated protein 4; VEGFR, vascular endothelial growth factor receptor; ALL, acute lymphoblastic leukemia; MRD, minimal residual lesions; NSCLC, non-small cell lung cancer; MCC, Merkel cell carcinoma; RCC, renal cell carcinoma*.

A phase III clinical trial (ClinicalTrials.gov number: NCT02231749) has demonstrated that combined immunotherapy can improve the survival rate of renal cell carcinoma. Compared with the use of the tyrosine kinase inhibitor sunitinib, a monoclonal antibody drug, the combination of nivolumab and ipilimumab in the treatment of melanoma, increased the overall survival rate to 18 months in patients with high-risk renal cell carcinoma (12, 13). A second-stage study (ClinicalTrials.gov number: NCT02320058) showed that the complete response rate and partial response rate were 26 and 30%, respectively, after treatment with nivolumab and ipilimumab and that 82.8% of patients were alive 9 months later (14).

In the past 2 years, the most popular chimeric antigen receptor (CAR) T cell immunotherapy has mainly focused on targeting CD19 in lymphoma. FDA-approved CAR-T cell therapies such as tisagenlecleucel (Kymriah, CTL019) and axicabtagene ciloleucel (Yescarta, KET-C10) (ClinicalTrials.gov number: NCT03123939) have shown long-term benefits (15, 16). Anti-CD19 CAR-T cell therapy is a promising option for patients with relapsed and refractory B cell acute lymphoblastic leukemia (15).

Unlike systemic chemotherapy, targeted drug therapies make use of specific gene mutations in cancer cells, unlike systemic chemotherapy. For example, osimertinib, a new epidermal growth factor receptor inhibitor (ClinicalTrials.gov number: NCT01802632), can delay the progression of lung cancer in response to drug resistance mutations (17). Osimertinib has become the preferred initial treatment for some non-small-cell lung cancer patients with epidermal growth factor receptor mutations (18). A targeted therapy for human epidermal growth factor receptor-2 (HER2)/neu mutation, trastuzumab, was developed to treat breast cancer (19). Abemaciclib (Verzenio) is a novel drug (ClinicalTrials.gov number: NCT02102490, NCT02107703, and NCT02246621) that inhibits the activity of the CDK4/6 (cyclin-dependent kinases 4/6) protein, which regulates the cell division rate, rather than targeting specific gene mutations (20–22).

## Engineered T Cell Therapy

**Figure 1:**
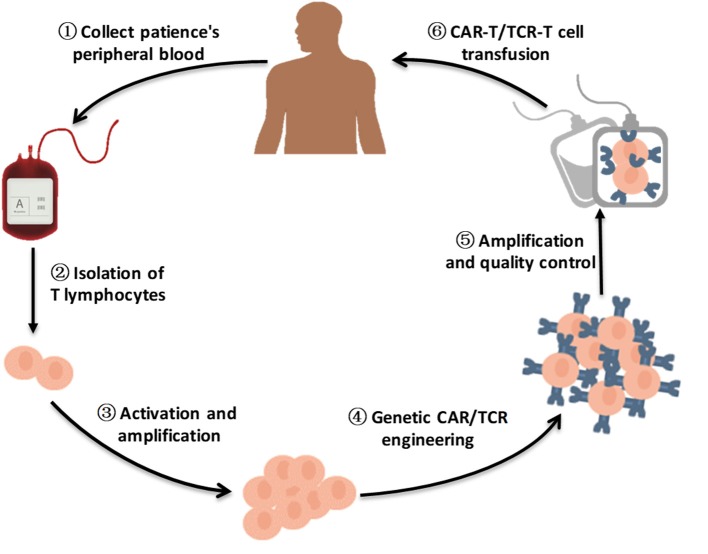
A brief flow chart of engineered-T cell therapy. A sufficient amount of blood is drawn from patients to obtain enough peripheral blood mononeuclear cells (PBMCs) for engineered T cell manufacturing. The T cells are purified from patients PBMCs. After activation and amplification *in vitro*, T cells are modified by viral vector transfection, such as lentivirus transfection or retrovirus transfection, to express specific CARs/TCRs on the T cell surface. Following amplification and quality control, CAR-T cells/TCR-T cells are infused into the patient body to improve antitumor ability.

Early adoptive immunotherapy mainly transfuses autologous or allogeneic tumor-responsive T cells back into the patient's body to attack the patient's tumors. This method has been successful (6, 23, 24). However, this therapy has not been widely used, mainly because of the small number of invasive lymphocytes and the fact that it cannot improve the anti-tumor ability of the body's autoimmune system (6). T cell genetic engineering therapies can overcome the challenges of low survival and migration of T cells and immune escape to a certain extent (25, 26). T cells are extracted from the patient's blood and genetically modified to encode receptors that recognize cancer-specific antigens (Figure 1). Additional genes, such as those encoding cytokines, can also be modified to prolong survival and promote T cell penetration into cancer tissue (26). CAR-T cell therapy and T cell receptor (TCR)-T cell therapy, as the latest and most effective immunotherapy technologies, have been widely studied in recent years. Clinical research regarding therapies using genetically engineered T cells has shown remarkable success.

## CAR-T Cell Therapy

CAR-T cell therapy is a novel tumor immunotherapy technique for cancer treatment (17, 27–29). The efficacy of CAR-T cells for the treatment of acute B lymphocytic leukemia has been widely recognized, and several clinical trials using CAR-T cell therapy in the treatment of various types of tumors have been reported (17, 27–29). This approach is also more specific and more customizable because the antigen-binding domain can be changed to target various tumor targets (17, 27–29). Moreover, CAR-T cells can also establish memory in patients with advanced leukemia (30).

**Figure 2:**
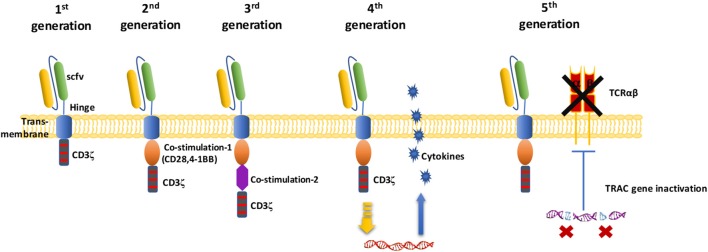
Schematic diagram of the CAR-T cell structure. In the first generation of CARs, there was only one intracellular signal component CD3ζ. The second generation of CAR added one costimulatory molecule on the basis of the first generation. Based on the second generation of CARs, the third generation of CAR added another costimulatory molecule. Fourth-generation of CAR T cells can activate the downstream transcription factor to induce cytokine production after the CAR recognizes the target antigens. The fifth-generation of CARs, based on the second generation, uses gene editing to inactivate the TRAC gene, leading to the removal of the TCR alpha and beta chains.

A CAR is the core component of CAR-T cells, making T cells MHC unrestricted. This phenomenon enables the modified T cells to recognize more extensive targets than the natural TCR on T cell surface and is not restricted by major histocompatibility complex (MHC) molecules. A CAR is a recombinant receptor with both tumor-antigen-binding and T cell-activating functions (Figure 2) (31, 32). The extracellular domain is a single-chain variable antibody domain (scFv), which can recognize specific antigens of tumors (25). The heavy-chain variable region (VH) and light-chain variable region (VL) of antibodies are linked by a small segment of polypeptide. Via the scFv, CAR-T cells can directly recognize and bind to tumor-specific antigens (25, 33). The hinge domain is composed of immunoglobulin superfamily members, such as CD8, CD28, or IgG, which play a role in signal transduction (25). The intracellular signal transduction region is mainly composed of the CD3ζ chain of the TCR (25). In addition to intracellular signaling domains, costimulatory molecules such as CD28 or 4-1BB (CD137) can improve cell proliferation and survival time *in vivo* and enhance the anti-tumor activity of CAR-T cells (25, 34). CAR-T cells directly recognize tumor surface antigens, and are not restricted by MHC class. When CAR-T cells bind to tumor surface antigen, they proliferate and kill tumor cells.

The activation of T cells mediated by first-generation CARs is accomplished through the tyrosine activation motif on the CD3ζ chain (Figure 2) or FcRγ (25, 35, 36). The CD3ζ chain can provide signals for T cell activation and target cell lysis, regulation of IL-2 secretion, and anti-tumor activity *in vivo* (36). However, the anti-tumor activity of first-generation CAR-modified T cells is limited *in vivo*, and this decreased T cell proliferation ultimately leads to T cell apoptosis (35, 36). Second-generation CARs incorporate an additional costimulatory signal. Experiments show that this signal amplifies the original “signal 1” derived from the TCR/CD3 complex and increases T cell proliferation and cytokine secretion, promoting the secretion of anti-apoptotic proteins (37). A commonly used costimulatory molecule is CD28 or CD137(4-1BB). To further improve the design of CARs, many research groups have focused on the development of third-generation CARs, which include not only CD3ζ and one costimulatory domain but also an additional costimulatory signal (34). Based on these second or third-generation CARs, fourth-generations CAR-T cells coexpress some key cytokines or costimulatory ligands, such as IL-12, IL-15, and IL-7, or suicide genes, which significantly enhance the expansion activity of T cells. For fifth generation CAR-T cells, it has been proposed to knock out the human leukocyte antigen (HLA) and TCR genes of T cells obtained from healthy donors to avoid host immune rejection or graft-vs.-host disease against transplanted CAR-T cells (3, 18, 38). Because it does not need to be modified according to the patient, this strategy can be used for the treatment of multiple patients (18).

Different researchers have used different targets and costimulatory signals to compare the results of second-generation and third-generation CARs. Some studies have reported that recombinant T cells expressing third-generation CARs have significantly increased anti-tumor activity, survival, and cytokine release abilities (34). It is worth noting that the above differences are conclusions obtained from only ex vivo mice experiments, and there are no *in vivo* comparisons of second-generation and third-generation CARs. The difference between the two generations of CARs may originate not only from the signal transduction domain but also from the extracellular antigen-binding domain (scFv), the transfection method used for the recombinant T cells (Lentivirus vs. Retrovirus), and the transfusion mode of recombinant T cells (intravenous transfusion vs. peritoneal infusion vs. intratumor infusion).

### CAR-T Cell Therapy Process

CAR-T cell therapy is a revolutionary targeted immunotherapy (17, 27–29). It necessitates modification of patient T cells outside the body and retransfusion of these cells back into the human body to fight the target cancer cells. The typical CAR-T cell production process is divided into five steps (Figure 1) (25). The first step is to isolate T cells from cancer patients. The second step is to modify the T cells with CARs so that the T cells can simultaneously recognize tumor cells and activate T cells, creating CAR-T cells (6, 25). In the third step, CAR-T cells are cultured ex vivo and stimulated by cytokines to produce a large number of CAR-T cells (25). The fourth step is to transfuse the expanded CAR-T cells back into the patient at an appropriate dose (25). Finally, patients need to be closely monitored, especially to monitor and control severe physical reactions in the following few days (6). The whole process lasts approximately 3 weeks, and the preparation of CAR-T cells requires approximately 2 weeks, making the cell preparation step the most time-consuming step (25).

CAR-T cells are expanded ex vivo and frozen for future administration. Patients are given preconditioning chemotherapy (6). Following tumor burden reassessment, CAR-T cells are infused. When the antigen-binding domain recognizes malignant antigen, it stimulates the downstream activation signal and produces specific killing effects. The use of such CAR-T cell therapies in B cell lymphoma/leukemia in the clinic has achieved complete remission in a number of relapsed and refractory advanced patients (15).

#### Recruited Patients

To lay a foundation for the application of CAR-T cell therapy, clinical trials recruit suitable patients, and they must satisfy certain conditions. Patients aged older than 75 years or younger than 1 year will be detrimental to clinical trials, and the survival time should be at least 3 or 6 months. The recruited patients are usually relapsed or refractory, or they have experienced chemotherapy failure, bone marrow transplantation failure, or autologous, allogeneic hemopoietic stem cell transplantation failure, or have been unable to find an effective treatment. Although patients are widely recruited, some patients are excluded, such as those who have clinically significant cardiovascular disease or those who are pregnant or lactating. Patients who have participated in any other clinical trials in the past 30 days are excluded. Additionally, patients with any type of primary immunodeficiency are excluded from the clinical studies. Other symptoms are not applicable for CAR-T cell therapy because they may increase patient risk or interfere with clinical test results. The main goal of trials is to evaluate the safety, effectiveness, and feasibility of CAR-T cell immunotherapy.

#### Pretreatment of the Patient

In addition to killing cancer cells directly, the use of some chemotherapeutic drugs, such as fludarabine (FA) and cyclophosphamide (CTX), as pretreatment regimens before immunotherapy, may produce synergistic immunobiological effects, thereby enhancing the effects of anti-tumor immunotherapy (39). Pretreatment chemotherapy can remove existing lymphocytes in lymphoid tissue or bone marrow tissue, establish implantation space for newly implanted antitumor immune cells, and induce the production of bone marrow cytokines, thus promoting the recovery and proliferation of immune cells (28, 40). CTX is the most commonly used drug for pretreatment in cancer immunotherapy. In addition, other commonly used pretreatment chemotherapy drugs include FA, gemcitabine (GEM), lenalidomide, and docetaxel (DTX). For example, when FRα (Folate receptor α) CAR-T cells were used to treat patients with end-stage ovarian cancer, the pretreatment regimen was CTX 300 mg/m^2^/day for three consecutive days and FA 30 mg/m^2^/day for three consecutive days (41). For melanoma patients, Deniger et al. of the National Cancer Institute (NCI) formulated a routine regimen of 60 mg/kg/day CTX for 2 days and 25 mg/m^2^/day FA for 5 days (42). However, the dosage, mode, and sequence of pretreatment still need further optimization and verification by more clinical trials.

#### Injection Dose

In clinical treatment, CAR-T cells are injected at a wide range of doses, sometimes with dose-increasing regimens. Some reports suggest that there is a correlation between the dose of CAR-T cells injected and the incidence and severity of the cytokine release storms (CRS) (43). The total number of CAR-T cells ranges from 1 × 10^5^/kg to 1 × 10^10^/kg CAR-T cells or more. Sometimes, to investigate the patient's tolerance, the total number of cells is injected over three courses, 10% on the first day, 30% on the second day, and 60% on the third day (44). One patient with chronic lymphoblastic leukemia who was treated with CAR-T cell therapy has been cancer free for 5 years (45). More importantly, that study found that the minimum cell number required for CAR-T cell therapy to exert its anticancer effects was only one cell (45).

### Success and Advantages of CAR-T Cell Therapy

Emily Whitehead was the first pediatric ALL patient to receive CAR-T cell treatment. The University of Pennsylvania CAR-T cell clinical trial completely cured Emily's leukemia, and Emily has become a spokesperson for CAR-T cells. Here, we present three recent successful cases to illustrate the potential of CAR-T cell immunotherapy in cancer treatment. In a clinical trial for children and young adults with cancer, none of whom had responses to standard therapy, a CAR-T cell therapy called tisagenlecleucel (Kymriah) was successful in 52 of 63 patients. In the study, three out of every four patients did not relapse after 6 months (46). According to the results of this study, FDA approved tisagenlecleucel in August 2017 for the treatment of relapsed or refractory B-cell precursor ALL in patients ≤25 years old.

Another study used CAR-T cell therapy to treat refractory large B-cell lymphoma. The CAR-T cell therapy used in this study is called axicabtagene ciloleucel (Yescarta) (28). Immunotherapy slowed or stopped the growth of cancer in 82% of patients, and more than half (54%) of the cancers disappeared completely (28). Nearly 15.4 months later, approximately 40% of patients still showed no signs of cancer. In October 2017, the FDA approved axicabtagene ciloleucel for the treatment of adults with relapsed or refractory large B-cell lymphoma, including diffuse large B-cell lymphoma (DLBCL), after two or more lines of systemic therapy.

Multiple myeloma is an incurable disease, and only approximately half of patients are still alive 5 years after diagnosis. Early clinical trials presented at the 2017 American Society of Clinical Oncology (ASCO) annual meeting showed that CAR-T cell therapy using B cell maturation antigen (BCMA) as a biomarker could prevent the development of multiple myeloma. The study included 35 patients with multiple myeloma who relapsed after treatment or were resistant to treatment. Of these 35 patients, 74% experienced a complete response of multiple myeloma after receiving BCMA CAR-T cells (47).

CAR-T cells are not restricted by MHC molecules. They specifically recognize antigens and kill tumor cells more effectively. Tumor infiltrating lymphocytes (TILs) and TCRs can recognize only the antigens presented by specific MHC molecules and tumors may escape immune surveillance due to downregulation or mutation of MHC molecules in tumor cells, resulting in clinical limitations (47).

### Failures and Challenges of CAR-T Cell Therapy

The first third-generation CAR-T cell clinical studies included HER2-targeted CAR-T cell therapy and indications for metastatic melanoma. The NCI chose to make use of an scFv targeting HER2 in a CAR-T cell for therapy for HER2+ melanoma (48). The first patient received a high concentration of CAR-T cells, with a total dose of 1 × 10^11^ cells. After treatment, the patient experienced extreme pain within a few minutes and soon fell into a coma. Doctors administered a high-dose hormone intervention, and the patient died 5 days later. Subsequently, CAR-T cells were found throughout the patient's body, and the lungs were the most seriously infiltrated organ. Further analysis may find that HER2 expression in pulmonary epithelial cells was the main cause of this fatal lung T cell infiltration (48).

Some types of immunotherapy can attack cancer or slow its spread to other parts of the body. Others make it easier for the immune system to destroy cancer cells, but immunotherapy sometimes leads to the immune system attacking healthy cells, resulting in side effects. Different types of immunotherapy can cause different side effects. The occurrence of many side effects depends on the type of treatment, the type and location of the cancer, and the patient's overall health. Skin redness, blistering, and dryness are common reactions associated with immunotherapy. Fatigue, fever, chills, weakness, nausea, vomiting, dizziness, body pain, and hypertension or hypotension are also side effects of immunotherapy. This new approach to cancer treatment is powerful, and serious risks need to be considered before starting treatment. In particular, there are always possible side effects of treatment, including CRS and neurological problems.

#### CRS

CRS is caused by the production of a large number of inflammatory molecules by CAR-T cells. CRS can cause long-term fever, hypotension, dyspnea, and organ problems. Severe CRS can be a life-threatening problem, requiring intensive medical care, including the use of ventilators, drugs to increase blood pressure, and antiepilepsy drugs. The FDA approved intravenous injection of tocilizumab (Actemra), an IL-6 receptor inhibitor, for the treatment of CAR-T cell-induced severe or life-threatening CRS in patients 2 years of age and older. CRS is the most frequently mentioned adverse reaction in CAR-T cell treatment. After infusion of CAR-T cells, a systemic inflammatory reaction caused by a rapid rise in cytokines such as IL-1 and IL-6 was observed in a mouse model (49, 50). CRS usually occurs within 2 days after CAR-T cell infusion, and the worst part of the reaction occurs within 1–2 weeks after CAR-T cell infusion (51). It is worth noting that in the pathophysiological process of CRS, not only activated CAR-T cells but also monocytes, macrophages, and dendritic cells participate in the synthesis and release of cytokines, which leads to the corresponding clinical symptoms (50). Studies have shown that patients with massive lesions, patients with a high tumor burden, patients with complications, and patients with CRS after 3 days of CAR-T cell infusion are prone to severe CRS (50). Therefore, treatment evaluation before CAR-T cell infusion is of great significance for CRS control and prognostication. For tocilizumab, a dosage of 4 to 8 mg/kg should be infused intravenously (51). It should be noted that tocilizumab cannot effectively improve the neurotoxicity of CRS due to the blood–brain barrier (50). Methylprednisolone has been used in patients with severe CRS who did not receive tocilizumab.

#### Neurotoxicity

The overall incidence of neurotoxicity is 40% (52). The most common symptoms include decreased consciousness, confusion, seizures, and brain edema. Neurotoxicity can occur alone or simultaneously with CRS (31). Mild clinical signs can spontaneously regress within a few days. Severe symptoms may require treatment with single agents or combination with dexamethasone (10 mg intravenously or orally) (51, 53). Strategies to reduce CRS and neurotoxicity fall into two categories: preventive strategies aimed at reducing the occurrence of severe toxicity and remedial strategies aimed at minimizing toxicity once lethal toxicity occurs. Preventive strategies include reducing the burden of cancer by chemotherapy and reducing the dose of CAR-T cells in patients with high cancer burden before receiving CAR-T cell infusion (52). Without affecting the treatment of CAR-T cells, early intervention with tocilizumab and dexamethasone seems to reduce the incidence of severe CRS (51).

#### On-Target–Off-Tumor Toxicity

The ideal target antigen is tumor-specific and expressed only on the surface of cancer cells. Unfortunately, most of the antigens expressed in tumors are not specific to tumors; For example, most CARs target tumor-associated antigens, but this often leads to the possibility of mistargeting. Additionally, attention should be paid to the selection of secretory antigens to avoid the possibility of mistargeting by CAR-T cells. As long as the target is not 100% tumor-specific, there will be an off-target effect, which is the main source of CAR-T cell side effects. This means that these targets are expressed not only in cancer tissues but also in normal tissue (52). Specific antigens expressed only on the surface of cancer cells can be selected, or multiple antigen complex CAR structures can be designed to identify antigens. The best example of remedial strategies is the addition of suicide or elimination genes to CAR-T cells, which can be activated to selectively deplete CAR-T cells in the event of severe toxicity (54). Thus, “on-target–off-tumor” toxicity can be efficiently avoided.

#### Exhaustion of CAR-T Cells

Because of T cell exhaustion, T cells entering solid tumors may stop working. Coexpression of some cytokines, such as IL-15, promotes T cell proliferation and persistence in a mouse model (55). The Nr4a (nuclear receptor transcription factors) family of transcription factors plays a prominent role in regulating genes related to T cell exhaustion (56). Scientists have used melanoma mouse models to demonstrate that treatment with CAR-T cells lacking these Nr4a transcription factors can reduce tumor sizes and improve survival in mice (57). These results suggest that the nuclear factor of activated T cells (NFAT) and Nr4a proteins are helpful in resisting T cell exhaustion in cancer and are “exhaustion markers” of T cells in a mouse model (57). However, the results of this experiment cannot be used in clinical practice because the consequences of editing multiple genes in human cells are unknown. Understanding the roles of Nr4a transcription factors provides cancer researchers with new targets for designing better therapies. The molecular design of CARs also has a significant effect on the proliferation and persistence of CAR-T cells. A CAR carrying the inducible costimulatory molecule (ICOS) costimulatory domain (ICOS-CAR) shows an improved ability to maintain the persistence of CD4+ T cells in a mouse model costimulatory (58). By injecting a mixture of CD4+ and CD8+ T cells expressing different CARs into mice at a ratio of 1:1, researchers found that CD4+ T cells expressing an ICOS-CAR not only increased their own persistence but also increased the persistence of the CD8+ T cells (58). The anticancer effect of this cell combination was also strongest in mice with non-small-cell lung cancer. When ICOS and 4-1BB costimulatory domains were added to the intracellular costimulatory domain of a third-generation CAR-T cell (ICOSBBz-CAR-T cell), and both CD4+ and CD8+ T cells expressed this ICOSBBz-CAR, the resulting T cells not only had better persistence but also had a better anticancer effect than a combination of CD4+ T cells expressing ICOS-CAR and CD8+ T cells expressing BBz-CAR (58). The expression level of ICOSBBz-CAR on the T cell surface was lower than that of ICOS-CAR or BBz-CAR. A possible reason for this finding is that the expression of the CAR was too high, which can cause T cells to be continuously activated, leading to their early depletion.

#### Tumor Escape

Although the patient response rate to CAR-T cells is very high, many patients treated with CAR-T cells will have disease recurrence. There are many reasons for this recurrence, such as antigen downregulation on tumor cells or low antigen levels. The mechanism of cancer evasion is closely related to the TME. The TME can influence the heterogeneity of tumors and play a key role in the subsequent development and metastasis of tumors (59). Mechanisms of immune escape include alterated of the expression of G1 regulatory proteins; production of inhibitors such as IL-10, transforming growth factor-beta (TGF-β), and indoleamine 2,3-dioxygenase (IDO); overexpression of immunosuppressive receptors such as PD-L1; and recruitment of Tregs (60). Therefore, combination therapies with immunological checkpoint inhibitors (such as a PD-1/PD-L1 antibody) should be adopted.

#### Treatment for Solid Tumors

The unprecedented success of CAR-T cell therapy in the treatment of hematological tumors has inspired people to extend this technology to solid tumors. Increasing numbers of clinical trials using CAR-T cell therapy for solid tumors have been carried out. However, the results of clinical trials are often unsatisfactory. The therapeutic effect of CAR-T cell therapy for solid tumors is greatly inferior to that of CAR-T therapy for hematological tumors and is often accompanied by toxicities (61). Hematological tumor cells are dispersed, and solid tumors usually form solid masses in certain organs early in the cancer process, which not only causes many obstacles for the recruitment of immune cells to the affected area but also results in the accumulation of many kinds of immunosuppressive cells and molecules (60). The antigens of hematological tumors are often specific and not expressed in other normal tissues, while the antigens of solid tumors are generally expressed in small amounts in other locations, such as the heart, lung, and liver, leading to mistargeted effects after treatment.

#### Encephaledema

In May 2016, one patient treated with JCAR015 died of cerebral edema, and two died of the same symptoms in July, so the experiment was suspended (61). Juno's researchers explained that the pretreatment scheme used CTX and FA and reported that FA would be eliminated from the pretreatment regimen in the subsequent attempt. Two more patients died of brain edema after the trial was restarted, and the trial was suspended again (61). In fact, the problem was not FA. It may have occurred because the costimulatory signal of CD28 is too strong, resulting in immune side effects and leading to brain edema. While 4-1BB is not as strong as CD28 as a costimulatory molecule, it provides late-acting signals to regulate T cell poliferation and survival (62). In the late stage of the immune response, T cells express inhibitory receptors such as CTLA-4, while CD28 expression is downregulated, leading to T cell loss of the second activation signal of the CD28-B7 pathway. However, 4-1BB is still expressed in the late stage of the T cell response, indicating that it persists longer.

#### The Cost of CAR-T Cell Therapy

The two available CAR-T cell therapies are currently very expensive. Novartis's CAR-T cell therapy, Kymriah, costs an average of ~$510,963, and Yescarta costs an average of approximately $402,647 (63, 64). If the patient does not experience an effect after CAR-T treatment for 1 month, the medical expenses can be waived. It takes approximately half a month to 1 month to make a specific CAR-T cell product for use in patients. Many enterprises are now studying more efficient and stable production strategies for CAR-T cells. For example, ThermoGenesis, a U.S. automation equipment company, has developed a platform called CAR-TXpress™ that specializes in producing CAR-T cells. The production cost of CAR-T cells can be greatly reduced. CAR-T cells are produced faster, and the quality of the cells is better. Thus, companies not only can use higher-quality cells for clinical research, allowing faster development of CAR-T cell therapies for more diseases, but also can achieve faster commercialization, making CAR-T cell therapies more readily available on the market (65). Because the cost of CAR-T cell immunotherapy is very high, many patients with leukemia who cannot afford such therapies are excluded from treatment, but with the development of this production strategy and the implementation of a series of national medical policies, it is believed that an increasing number of patients will benefit from this therapy.

### Current Clinical Target of CAR-T Cell Therapy in Hematological Malignancies

**Figure 3:**
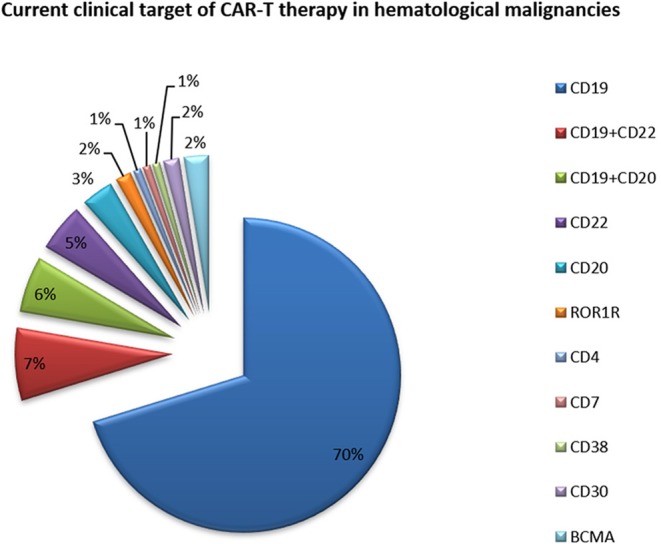
Current clinical targets for CAR-T treatment of hematologic malignancies. The pie chart is based on the statistical result of CAR-T clinical trials for hematologic malignancies registered on ClinicalTrials.gov.

The expression of CD molecules as surface markers can be used for the identification of cell types. Several clinical studies using CD19 as the target of CAR-T cells have been registered. In addition to CD19, which is the most widely reported target, there are many tumor-related targets that have entered phase I and phase II clinical trails. In currently registered clinical trials and published research results, B cell maturation antigen (BCMA) has been presented as a particularly important target. At present, there are many targets involved in clinical CAR-T cell projects. Current clinical trials of CAR-T cell therapies mainly focus on CD19, CD20, CD22, GPC3 (Glypican-3), BCMA, and other promising targets (Table 2 and Figure 3).

**Table 2:** Current clinical targets of CAR-T therapy for hematological malignancies (ClinicalTrials.gov).

**Target**	**Disease**	**Stage**	**Phase**	**NCT number**	**Country**
CD19	Refractory CD19+ lymphoma and leukemia	Completed	I	NCT01626495	United States
	B-cell leukemia or lymphoma		I	NCT01029366	United States
	Refractory B cell malignancy		I/II	NCT02132624	Sweden
	B cell leukemia or lymphoma		I	NCT01593696	United States
	ALL		I	NCT01551043	United States
	Resistant or refractory CD19+ ALL		I	NCT02975687	China
	Resistant or refractory CD19+ ALL		II	NCT02030847	United States
CD19 + CD22	Relapsed or refractory lymphoma and leukemia	Recruiting	I/II	NCT03398967	China
	CD19 positive diffuse large B-cell lymphoma or B ALL		I	NCT03233854	United States
	CD19 +CD22+ leukemia		I	NCT03330691	United States
	Refractory and/or Recurrent B cell malignancies		I/II	NCT03098355	China
	Children or young adults with CD19 positive B ALL		I	NCT03241940	United States
	B-cell ALL		I/II	NCT03614858	China
	B-cell ALL		I/II	NCT03289455	United Kingdom
	B-cell hematologic malignancy		I/II	NCT02903810	China
	CD19/CD22-expressing B cell malignancies		I	NCT03448393	United States
CD19 + CD20	Relapsed or refractory lymphoma and leukemia	Recruiting	I/II	NCT03398967	China
CD22	CD22+ leukemia and lymphoma	Active	I	NCT03244306	United States
	Chemotherapy resistant or refractory ALL	Terminated	I	NCT02588456	United States
	B cell malignancies	Recruiting	I/II	NCT02935153	China
	B cell malignancies	Recruiting	I/II	NCT03262298	China
	ALL	Recruiting	I	NCT03620058	United States
	ALL	Recruiting	I	NCT02650414	United States
CD20	Refractory or relapsed B lymphocyte lymphoma	Recruiting	I	NCT03576807	China
	B cell malignancies	Recruiting	I/II	NCT02710149	China
	Relapsed or refractory B cell non-Hodgkin lymphomas	Recruiting	I/II	NCT03277729	United States
ROR1R	ROR1+ malignancies	Recruiting	I	NCT02706392	United States
	CLL	Withdrawn	I	NCT02194374	United States
CD4	CD4+ lymphoma and leukemia	Recruiting	I	NCT03829540	United States
CD7	High risk T cell malignancies	NR	I	NCT03690011	United States
CD38	Relapsed B-cell ALL after anti-CD19 CAR-T therapy	Recruiting	I/II	NCT03754764	China
CD30	Lymphocyte malignancies	Recruiting	I/II	NCT02958410	China
	Hodgkin lymphoma, adult	NA	–	NCT03914885	United States
BCMA	High-risk multiple myeloma	Recruiting	I/II	NCT03455972	China
	B-cell lymphoma and leukemia	Recruiting	I/II	NCT02954445	China

*CLL, chronic lymphocytic leukemia; ROR1R, receptor tyrosine kinase-like orphan receptor; NA, not available; NR, not yet recruiting*.

As of May 2018, 19 clinical applications for CAR-T cell projects from 13 enterprises had been accepted by the CDE (Drug evaluation center of state drug administration) in China; most of these applications targeted CD19. The clinical application of the LCAR-B38M CAR-T cell preparation submitted by Nanjing Legend was formally accepted by the CDE, and it became the first CAR-T cell product to be clinically accepted in China. This product is a BCMA-targeted CAR-T cell product that is mainly used for relapsed or refractory multiple myeloma. A total of 57 patients with multiple myeloma from the Second Affiliated Hospital of Xi'an Jiaotong University participated in the LCAR-B38M clinical trial (66). The overall response rate was 88%, and 68% of patients achieved a complete response.

The Department of Hematology, Jiangsu Institute of Hematology, China, conducted the first clinical study to treat relapsed/refractory acute lymphoblastic leukemia by sequential transfusion of two types of CAR-T cells: CD19-CAR-T cells and CD22-CAR-T cells (67). Infusion of mixed second-generation CAR-T cells is feasible and safe for patients with refractory/recurrent ALL. Combination therapy against multiple target antigens should be an effective way to overcome recurrence after antigen escape.

### Current Clinical Targets of CAR-T Cell Therapy in Solid Tumors

**Figure 4:**
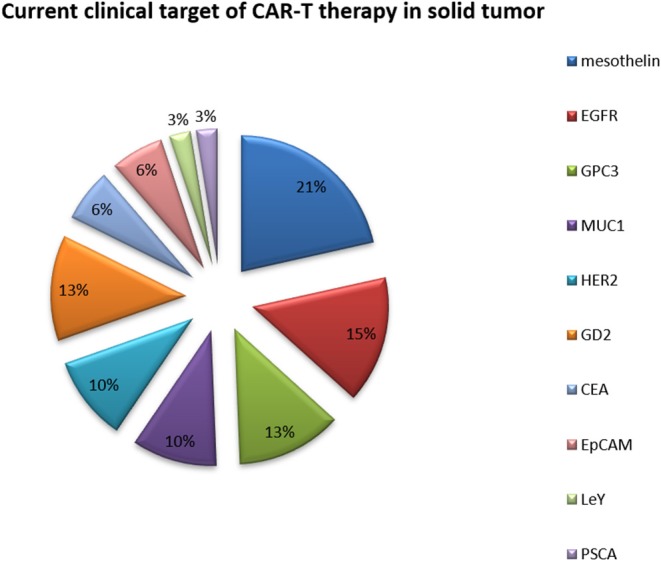
Current clinical targets for CAR-T therapy in solid tumors. The pie chart is based on the statistical result of CAR-T clinical trials for solid tumors registered on ClinicalTrials.gov.

Because of their relatively isolated physiological locations and special immunosuppressive microenvironments, immunotherapy is less effective for solid tumors than for hematological tumors (Table 3 and Figure 4) (68). Regulating the level of chemical flooding factors on the surface of T cells can encourage these cells to enter solid tumors. The immunosuppressive microenvironment of solid tumors is mainly caused by cytokines such as IL-6 and TGF-beta and immunosuppressive cells such as Tregs and myeloid-derived suppressor cells (MDSCs). The use of neutraling (blocking) antibodies against these cytokines and immunosuppressive cells can reverse the immunosuppressive microenvironment to some extent (69). Moreover, the use of nonspecific PD-1 and CTLA-4 antibodies to reverse T cell inhibition can also resolve the poor efficacy of T cell immunotherapy for solid tumors to a certain extent (68, 69).

**Table 3:** Current clinical targets of CAR-T therapy for solid tumors (ClinicalTrials.gov).

**Target**	**Disease**	**Stage**	**Phase**	**NCT number**	**Country**
Mesothelin	Recurrent or metastatic malignant tumors	Recruiting	I	NCT02930993	China
	Malignant pleural mesothelioma	Completed	I	NCT01355965	United States
	Metastatic pancreatic (ductal) adenocarcinoma	Completed	I	NCT02159716	United States
	Epithelial ovarian cancer	Completed	I	NCT01897415	United States
EGFR	EGFR+ advanced solid tumor	Recruiting	I/II	NCT03182816	China
	EGFR+ colorectal cancer	Recruiting	I/II	NCT03152435	China
	Metastatic colorectal cancer	NR	I	NCT03542799	China
	Recurrent or refractory pediatric CNS tumors	Recruiting	I	NCT03638167	United States
	Recurrent glioblastoma multiforme'	Recruiting	I	NCT02844062	China
GPC3	Advanced HCC	Completed	I/II	NCT03130712	China
	HCC	Recruiting	I	NCT02905188	United States
MUC1	Intrahepatic cholangiocarcinoma	Recruiting	I/II	NCT03633773	China
	NSCLC	Recruiting	I/II	NCT03525782	China
	Esophageal cancer	Recruiting	I/II	NCT03706326	China
HER2	Brain or leptomeningeal metastases	Recruiting	I	NCT03696030	United States
	HER2+ cancer	Recruiting	I/II	NCT02713984	China
	Breast cancer	Completed	I/II	NCT02547961	China
	HER2+ CNS tumors	Recruiting	I	NCT02442297	United States
	Recurrent/refractory pediatric CNS tumors	Recruiting	I	NCT03500991	United States
	Advanced solid tumors	Unknown	I/II	NCT01935843	China
GD2	High risk and/or relapsed/refractory neuroblastoma	Recruiting	I/II	NCT03373097	Italy
	Glioma	Recruiting	I	NCT02761915	United Kingdom
	Glioma	Completed	I/II	NCT03252171	China
CEA	Metastatic pancreatic carcinoma	Recruiting	I	NCT03818165	United States
	Liver metastases	Completed	I	NCT02416466	United States
EpCAM	Nasopharyngeal carcinoma	Recruiting	I	NCT02915445	China
	Advanced gastric cancer with peritoneal metastasis	Recruiting	I	NCT03563326	China
LeY	Advanced cancer	Recruiting	I	NCT03851146	Australia
	Myeloid malignancies	Recruiting	I/II	NCT02958384	China
PSCA	Metastatic castration resistant prostate cancer	Recruiting	I	NCT03873805	United States

*CNS, central nervous system; EGFR, epidermal growth factor receptor; GD2, ganglioside; CEA, carcino-embryonic antigen; EpCAM, epithelial cell adhesion molecule; Ley, Lewis Y antigen; PSCA, prostate stem cell antigen; HCC, hepatocellular carcinoma; NR, not yet recruiting*.

### Published Clinical Targets of CAR-T Cell Therapy in Hematological Malignancies

CAR-T cell immunotherapy has been proven to be a breakthrough in the treatment of hematological tumors, showing good targeting, lethality, and persistence (Table 4). The two CAR-T cell products (17, 29) approved by Novartis and Kite Pharma are suitable for acute lymphoblastic leukemia and B cell lymphoma, respectively, and their application is being investigated in blood cancers (17, 29). The application of CAR-T cell therapy in solid tumors has been relatively slow. Statistics show that approximately 75% of CAR-T cell clinical trials in the world are used for leukemia, lymphoma, and other blood cancers, and only a small number of CAR-T cell projects target solid tumors such as liver cancer and lung cancer.

**Table 4:** Published clinical targets of CAR-T therapy for hematological malignancies.

**Target**	**Disease**	**Costimulatory domain**	**Vector**	**Pretreatment**	**Dose**	**No. of patients**	**Phage**	**Response**	**Country**	**References**
CD19	B-ALL	CD28	Lentivirus	FLU, CY	5 × 10^4^−1 × 10^6^/kg	3	I	Grade 2 or 3 CRS;	China	(70)
CD19	B-ALL	4-1BB	Lentivirus	FLU, CY	Total dose of 1.19 × 10^6^/kg	2	–	Acute GVHD	China	(71)
CD19	B-ALL	CD28 + 4-1BB	Lentivirus	CE, FLU	2 × 10^5^−2 × 10^7^/kg	30	I/II	93% remission	United States	(72)
CD19	HL	4-1BB	Lentivirus	FLU, CY	Median dose of 1.56 × 10^7^/kg	18	I/II	2/18 grade ≥3 toxicities; 13/18 remission	China	(73)
CD19	CLL; DLBCL	CD28	Retrovirus	FLU, CY	1 × 10^6^/kg	15	–	80% remission; 1 died	United States	(74)
CD19	B-ALL	4-1BB	Lentivirus		7.6 × 10^5^−2.06 × 10^7^/kg	30	I/II	90% remission; 27% seriously CRS	United States	(53)
CD19	B-ALL	4-1BB	Lentivirus	FLU, CY	1.4 × 10^7^−1.1 × 10^9^/person	14	I	57% remission; 4/14 complete remission	United States	(75)
CD19	B-ALL	CD28	Retrovirus	CY	Total dose of 3 × 10^6^/kg	16	I	88% regression;	United States	(76)
CD19	NHL	CD28 + 4-1BB	Lentivirus	CY, VP	2 × 10^6^−2 × 10^7^/kg	32	I	13/32 grade ≥3 CRS; 72% remission	United States	(77)
CD20	DLBCL	4-1BB	Lentivirus	CY, VCR	1 × 10^7^/person	7	–	5 tumor regression	China	(78)
CD22	B-ALL	4-1BB	Lentivirus	CY, FLU	3 × 10^5^−3 × 10^6^/kg	21	I	73% complete remission; 8 relapsed	United States	(79)
CD30	HL	CD28	Retrovirus	–	2 × 10^7^-2 × 10^8^/m^2^	9	I	1/9 complete remission	United States	(80)
LeY	AML	CD28	Retrovirus	FLU	5 × 10^8^−1.3 × 10^9^ /person	5	I	1 relapse; 1 long-term remission	Australia	(81)

*B-ALL, B-cell precursor acute lymphoblastic leukemia; FLU, fludarabine; CY, cyclophosphamide; CNS, cytokine release syndrome; GVHD, graft-vs.-host disease; CE, cyclophosphamide and etoposide; HL, Hodgkin's lymphoma; DLBCL, diffuse large B cell lymphoma; VP, etoposide; VCR, vincristine; AML, acute myelocytic leukemia*.

The FDA announced approval of Kite Pharma's CAR-T cell therapy Yescarta for adult patients with specific types of large B cell lymphoma in October 2017. These patients had received at least two other treatments, but no remissions occurred. It is worth mentioning that this is the first non-Hodgkin lymphoma-specific CAR-T cell therapy approved by the FDA (17, 29) and the second approved CAR-T cell therapy. In a multicenter clinical trial, more than 100 adult patients were treated with Yescarta and showed surprising results: complete remissions (CRs) with Yescarta was observed in 51% of patients.

Researchers from the University of Texas M.D. Anderson Cancer Center in Houston conducted a CAR-T cell study involving 111 patients who had a specific type of B cell lymphoma and relapsed after other treatments. In this study, the researchers collected T cells from patients, genetically modified them under laboratory conditions to target lymphoma cells, and eventually transfused them back into the patients after amplification. After a median follow-up of 15.4 months, 42% of patients continued to have a response and 40% of patients had a complete response (28). As expected, 13% had grade 3 or higher CRS, which led to fever, and 28% had neurological disorders, among other adverse reactions (ClinicalTrials.gov number: NCT02348216).

A new CAR-T cell therapy targeting the surface molecule CD22 has been designed. A phase I trial of this CAR-T cell platform recruited 21 children and young adults (aged 7–30) with B-ALL who had relapsed or had not responded to previous treatments (79). Fifteen of these patients also received CAR-T cell therapy targeting CD19, but no significant improvements were achieved. These patients were given different doses of the CD22-targeting CAR-T cell therapy. A total of 73% patients achieved complete remission after receiving ≥1 × 10^6^ CD22 CAR-T cells (79).

### Published Clinical Targets of CAR-T Cell Therapy in Solid Tumors

There are many reasons for the remarkable progress of CAR-T cell therapy in hematological tumors and the slow progress in solid tumors (82). On the one hand, there are major differences between solid tumors and hematological tumors. The high heterogeneity makes treatment of solid tumors with CAR-T cell therapy much more difficult. Additionally, tumor antigens associated with solid tumors are expressed not only in tumor tissues but also in normal tissues. This poses another challenge in the treatment of solid tumors with CAR-T cells (61). In addition, the homing and activation of CAR-T cells in solid tumors are also challenging. How to make CAR-T cells accurately home from the peripheral blood to function at the tumor site needs to be solved urgently, but a single CAR-T cell therapy will not solve this problem. Moreover, CAR-T cells can coexpress some cytokines to enhance their killing activity and homing function, such as IL-2, IL-7, and chemokine (C-C motif) ligand 19 (CCL19) (Table 5).

**Table 5:** Published clinical targets of CAR-T therapy for solid tumors.

**Target**	**Disease**	**Costimulatory domain**	**Vector**	**Pretreatment**	**Dose**	**No. of patients**	**Phage**	**Response**	**Country**	**References**
EGFRvIII	Glioblastoma	4-1BB	Lentivirus	Temozolomide radiation	1–5 × 10^8^/person	10	I	5 remission	United States	(43)
EGFR	NSCLC	4-1BB	Lentivirus	CY, CDDP	4.5 × 10^6^−1.09 × 10^7^/kg	11	I/II	7/11 remission;	China	(83)
GD2	NB	NA	Retrovirus	–	2 × 10^7^−1 × 10^8^/m^2^	19	I	11/19 remission	United States	(84)
GD2	Metastatic melanoma	CD28 + OX40	Retrovirus	FLU, CY	1–2 × 10^7^/m^2^	4	I	4 partial response	South Australia	(85)
IL13Rα2	GBM	NA	DNA electroporation	–	10.6 × 10^8^/person	13	I	3 relapse	United States	(86)
IL13Rα2	GBM	4-1BB	Lentivirus	–	Total dose of 5.2–9.2 × 10^7^/person	1	I	1 relapse	United States	(87)
HER2	PNET; DSRCT	CD28	Retrovirus	Chemotherapy	1 × 10^4^−1 × 10^8^/m^2^	19	I/II	1 necrosis; 4/19 remission;	United States	(88)
CAIX	RCC	NA	Retrovirus	–	3 × 10^8^−2.1 × 10^9^/person	11	–	–	Netherlands	(89)
CAIX	RCC	NA	Retrovirus	–	6 × 10^8^−4 × 10^9^/person	12	–	–	Netherlands	(90)
Mesothelin	PAC; MPM	4-1BB	mRNA electroporation	–	Total dose of 1.2 × 10^9^/person	4	–	1 allergic reactions and cardiac arrest	United States	(91)
Mesothelin	MPM; PDA	4-1BB	mRNA electroporation	Chemotherapy	4.4 × 10^9^ of one patient; 3.1 × 10^9^/m^2^ of another	2	I	1 partial response	United States	(92)
MUC1	Seminal vesicle cancer	CD28 + 4-1BB	Lentivirus	–	Intratumoral injection of 5 × 10^5^ cells at two sites, respectively	1	I	no side effects	China	(93)
CEA	mCRC	CD28	Lentivirus	CY, FLU	2.5 × 10^7^-1.52 × 10^10^/person	10	I	70% remission	China	(44)
CEA	Liver metastases	CD28	Retrovirus	–	1 × 10^8^−3 × 10^10^/person	6	I	5 patients died of progressive disease;	United States	(94)
CEA	Adenocarcinoma; PMP	NA	–	FLU, CY	1 × 10^9^−5 × 10^10^/person	14	I	Transient acute respiratory toxicity	United Kingdom	(40)
BCMA	MM	CD28	Retrovirus	CY, FLU	3 × 10^5^−9 × 10^6^/kg	12	I	1 relapse after 17 weeks; 1 complete remission	United States	(95)
c-Met	Breast cancer	4-1BB	mRNA electroporation	–	3 × 10^7^−3 × 10^8^/person	6	–	Tumor necrosis at the injection site	United States	(96)
PSMA	PCa	NA	Retrovirus	Chemotherapy	1 × 10^9^−1 × 10^10^/person	5	I	2/5 partial remission	United States	(97)
TAG72	CRC	NA	Retrovirus	–	3−3.11 × 10^10^/person	16	I	Low-grade CRS	United States	(98)

*CDDP, cis-diamminedichoro platinum; NB, neuroblastoma; GBM, glomerular basement membrane; PNET, primitive neuroectodermal tumors; DSRCT, desmoplastic small round cell tumor; PAC, perennial allergic colitis; MPM, malignant pleural mesothelioma; PDA, patent ductus arteriosus; mCRC, metastatic colorectal cancer; PMP, pseudomyxoma peritonei; MM, multiple myeloma; PCa, prostate cancer; CRC, colorectal cancer; CAIX, carbonic anhydrase IX, c-Met, c-mesenchymal–epithelial transition; PSMA, prostate-specific membrane antigen; TAG72, tumor-associated glycoproteins-72; NA, not applicable. The first generation of CAR contains a CD3 signaling domain*.

Researchers from the Department of Neurosurgery, Perelman School of Medicine at the University of Pennsylvania used CAR-T cells to target the tumor-specific antigen EGFRvIII to treat patients (43). In this study, the authors found two major challenges with this technique: one problem is that the expression of EGFRvIII in the tumor tissues of patients is quite variable; the other is that the TME has a strong immunosuppressive effect, which will gradually become more concerning over the course of treatment. Therefore, the authors believe that it is necessary to search for new tumor antigens and drugs to overcome these immunosuppressive effects simultaneously to achieve the killing of tumor tissue. This also explains why CAR-T cell therapy is ineffective in treating gliomas: the immunosuppressive effects of the TME and the complexity of gene mutations (43).

T cells are picky about their environment and die within a few days without proper nutrients. In addition, tumor tissue releases self-defensive chemicals that block the normal functioning of T cells. Researchers at the Fred Hutchinson Cancer Research Center used a synthetic receptor “skeleton” to coat cancer-specific T cells and nutrients to kill tumor cells in mouse model (99). The results showed that the cytoskeleton coated T cells could effectively reduce tumor size in mice with pancreatic cancer and melanoma, and the therapeutic effect was stronger than that of injecting T cells alone. Of course, this approach is still a long way from clinical application (99).

### Future Improvements

Scientists have developed a variety of ways to improve the anticancer efficacy of CAR-T cell therapy. CAR-T cells were further modified to secrete proinflammatory cytokines, thus protecting CAR-T cells in the inhibitory TME. Modified CAR-T cells secreting IL-12 have achieved encouraging results in a mouse model (100). To improve the persistence of CAR-T cells, scientists constructed a dual-receptor CAR, which enables engineered T cells to express two artificial receptors, one for TAA recognition and the other for cytokine-mediated growth stimulation of T cells. CAR-T cells are subject to suppressive immune checkpoint signals in the TME, such as PD-L1 or CTLA-4. These inhibitory receptor–ligand interactions can be blocked by monoclonal antibodies, thus eliminating the inhibition of T cells. CAR-T cells targeting tumor vessels are being studied for the treatment of melanoma and renal cancer. The presence of angiogenic growth factors (such as VEGF) in the TME and the overexpression of their receptors in tumor cells are associated with poor prognosis and metastasis (101). One potential reason for the failure of CAR-T cell therapy relates to the persistence of CAR-T cells; strategies such as the replacement of the costimulatory domain of 4-1BB and increased production of cytokines that stimulate T cell proliferation and activation have potential.

New CAR-T targets are necessary for the treatment of patients with hematological malignancies that do not express CD19. In addition, some patients treated with CD19-CAR-T cell therapy will relapse due to mutations that allow tumor escape. Currently, the most promising targets for the treatment of hematological malignancies include CD22, CD20, ROR1, immunoglobulin kappa chain, BCMA, and CD138 (for plasma malignancies) as well as CD33, CD123, and LeY (for myeloid malignancy).

There are many gene-editing tools, including zinc finger nucleases (ZFNs), meganucleases, transcription activator-like (TAL) effector nucleases (TALENs), and the Clustered regularly interspaced short palindromic repeats-associated protein 9 (CRISPR-Cas9 nuclease) (38). These techniques have been successfully applied in the study of engineered T cells. Due to the rapid development of this field, universal CAR-T cells may eventually be widely used. The manufacturing process for universal CAR-T cell therapy is simpler, and it is expected to achieve faster and more inexpensive off-the-shelf cell therapy products.

Challenges in this process include the selection of target antigens, the management of toxicity, and the regulation of the immunosuppressive TME. Many researchers are actively developing CAR-T cell therapies to achieve the same success in other hematological malignancies and solid tumors.

## TCR-T Cell Therapy

**Figure 5:**
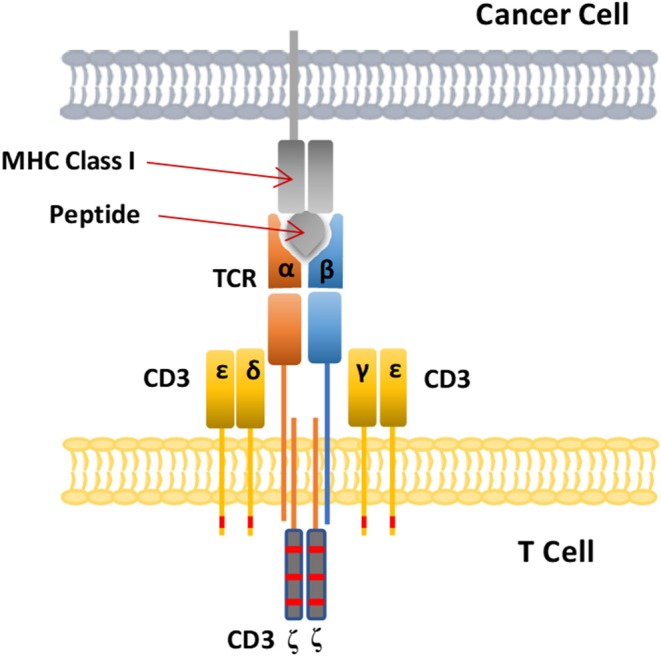
Schematic diagram of the CAR-T cell structure. The TCR complex is a heterodimer consisting of two different peptide chains, TCRα and TCRβ, and is surrounded by four CD3 chains. The MHC class I molecules present intracellular antigenic peptides of cancer cells for recognition bt the T cell receptor.

Both TCR and CAR-therapies modify the patient's own T lymphocytes *ex vivo* and then inject them back into the patient's body to kill cancer, but their mechanisms for recognizing antigens are quite different. TCRs use heterodimers consisting of alpha and beta peptide chains to recognize polypeptide fragments presented by MHC molecules (Figure 5) (102). CAR-T cells, on the other hand, use antibody fragments that bind to specific antigens on the surface of cancer cells. The target antigens identified by CAR-T cell therapy are all cell surface proteins, while TCR-T cell therapy can recognize intracellular antigen fragments presented by MHC molecules (102), so TCR-T cell therapy has a wider range of targets. However, TCR-T cell therapy is MHC restricted and depends on presentation by MHC molecules to recognize targets and activate T cell function, so these traits are also a disadvantage of TCR-T cell therapy.

Dr. Michael Steinmetz first transferred TCR genes from one T cell to another, giving the second T cell the same antigen specificity (103). This process is the origin of current TCR gene therapy. The TCR is a specific receptor and a characteristic marker on the surface of T cells. TCR-T cells are genetically engineered TCR products that can recognize specific antigens. The core goal behind TCR-T cell technology is to directly modify TCR binding to tumor antigens. The affinity of human TCRs to these antigens is relatively low, which makes it impossible to recognize and kill tumors effectively. The artificially designed high-affinity TCR is encoded in T cells by genetic engineering technology (54), which enhances both specificity recognition and affinity during the recognition of tumor cells by T cells.

The construction of high-affinity TCR-T cells requires the identification of specific targets. First, the polypeptides presented by cancer cells are identified, and then the polypeptides presented in normal tissues are screened out. After the target is confirmed, a TCR phage display library is established to screen TCRs with high affinity and specificity. A preclinical safety test is then performed to ensure minimal off-target effects and cross-reactivity.

In one report, after modifying several key amino acids, the resulting TCR had a greatly increased affinity for a common cancer antigen [tumor-associated antigen (TAA)], New York esophageal squamous cell carcinoma (NY-ESO-1), and this TCR can be used to attack NY-ESO-1-overexpressing cancers, such as multiple myeloma (104). In this clinical trial, 80% of patients with multiple myeloma had a good clinical response, and 70% of them had a complete or near-complete response (104).

### Advantages and Challenges of TCR-T Cell Therapy

Any antigen that can be presented by MHC molecules can be recognized by TCR-T cells, whether it is an intracellular or cell surface antigen or a neo-antigen produced by tumor cells after mutation. TCRs can recognize the internal molecules of cancer cells, and TCR-T cells can have greatly improved affinity for cancer cells through genetic engineering. Because they retain all the auxiliary molecules of the TCR signal transduction pathway, TCR-T cells can be fully activated when a small amount of antigen is present, resulting in a killing effect (26, 82).

Difficulties in the development of TCR-T cell technology include the selection of good targets, the search for specific TCRs, screening for optimal TCR affinity, safety evaluation, time, and cost. Target selection is one of the most important aspects (82). These problems have become barriers to TCR-T cell therapy, and finding a solution to these problems has become the key to determining the success of TCR-T cells.

Most TCR-T cell therapy targets are limited by MHC class. In addition, there is a risk of hybridization (mismatch) between exogenous and endogenous chains, which may induce harmful recognition of autoantigens, leading to graft-vs.-host disease. Increased TCR affinity poses a risk of false targeting, so TCR-T cell therapy should be applied carefully. TCR-T cell therapy has shown some therapeutic potential, but there are still many limitations, such as the matching of histocompatibility antigens and the need for antigens to be presented on the cell surface.

### Current Clinical Targets of TCR-T Cell Therapy in Hematological Malignancies

Specific targeted immunotherapy of leukemic stem cells is an ideal method for the treatment of malignant myeloid tumors (Table 6), but the appropriate epitopes are still unclear. The comparative proteomic characteristics of hematopoietic stem cells and progenitor cells from healthy stem cell donors and patients with acute myeloid leukemia may reveal differentially expressed proteins that can be used as surface markers or as substitutes for affected molecular pathways.

**Table 6:** Current clinical targets of TCR-T therapy for hematological malignancies (ClinicalTrials.gov).

**Target**	**Disease**	**Stage**	**Phase**	**NCT number**	**Country**
HA-1	Relapsed or refractory acute leukemia after donor stem cell	Recruiting	I	NCT03326921	United States
WT1	Myelodysplastic syndromes and acute myeloid leukemia patients	Completed	I/II	NCT02550535	Germany
WT1	Acute myeloid leukemia	Active	I/II	NCT02770820	United States
CMV	Hematological malignancies and CMV infection	Suspended	I	NCT02988258	United Kingdom

*CMV, cytomegalovirus; HA-1, a protein that present on the surface of blood cells; WT1, Wilms tumor 1*.

### Current Clinical Targets of TCR-T Cell Therapy in Solid Tumors

**Figure 6:**
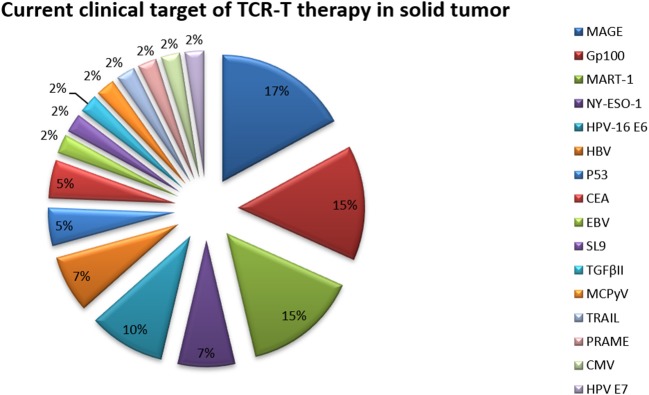
Current clinical targets for TCR-T therapy in solid tumors (ClinicalTrials.gov). The pie chart is based on the statistical result of TCR-T clinical trials for solid tumors registered on ClinicalTrials.gov.

T cells activated by TCRs can target most tumor-specific antigens, especially those antigens that are intracellular. Therefore, many TCR-T cell techniques have been applied in clinical trials of solid tumors (Table 7 and Figure 6). Because of their relatively isolated physiological locations and unique immunosuppressive microenvironments, immunotherapy is less effective for solid tumors than for hematological tumors. To overcome the isolated physiological locations of solid tumors, local injection of T cells into tumors was used in a mouse model (105). The effect from local injection is better than that achieved by systemic drug administration, such as injecting T cells into the cerebrospinal fluid in brain tumors.

**Table 7:** Current clinical targets of TCR-T therapy for solid tumors (ClinicalTrials.gov).

**Target**	**Disease**	**Stage**	**Phase**	**NCT number**	**Country**
MAGE	Solid and hematological malignancies;	Enrolling	–	NCT03391791	Canada
	Metastatic renal cancer and melanoma;	Terminated	I/II	NCT01273181	United States
Gp100	Metastatic melanoma;	Completed	II	NCT00923195	United States
	Malignant melanoma;	Terminated	II	NCT02889861	United Kingdom
MART-1	Skin metastatic melanoma	Completed	I	NCT00091104	United States
HPV-16 E6	HPV+ NHSCC or cervical cancer;	Recruiting	I	NCT03578406	China
	HPV-associated cancer;	Completed	I/II	NCT02280811	United States
NY-ESO-1	Ovarian, fallopian tube, or primary peritoneal cancer;	Recruiting	I	NCT03691376	United States
	Advanced NSCLC;	Recruiting	I	NCT03029273	China
	Sarcoma;	Recruiting	I	NCT03462316	China
HBV	Hepatocellular	Recruiting	I	NCT02719782	China
P53	Metastatic cancer that overexpresses p53	Completed	II	NCT00393029	United States
CEA	Metastatic cancer;	Terminated	I	NCT00923806	United States
HPV E7	Human papillomavirus-associated cancers	Recruiting	I/II	NCT02858310	United States
SL9	HIV	Completed	I	NCT00991224	United States
TGFβII	Metastatic colorectal cancer	Terminated	I/II	NCT03431311	Norway
MCPyV	Metastatic or unresectable Merkel cell cancer	Recruiting	I/II	NCT03747484	United States
TRAIL	Metastatic renal cancer	Terminated	I	NCT00923390	United States
PRAME	AML/MDS or metastatic uveal melanoma	Active	I/II	NCT02743611	United States
EBV	Recurrent or metastatic NPC	NR	II	NCT03648697	China
CMV	Hematological malignancies and CMV infection	Suspended	I	NCT02988258	United Kingdom
KRAS	KRAS G12V + tumor;	Recruiting	I/II	NCT03190941	United States
	KRAS G12D + tumor;	Recruiting	I/II	NCT03745326	United States

*NHSCC, head and neck squamous cell carcinoma; MDS, myelodysplastic syndromes; NPC, nasopharyngeal carcinoma; MAGE, melanoma antigen; Gp100, melanoma associated antigen; MART1, melanoma antigen recognized by T cells 1; HPV-16-E6, human papillomavirus-16 E6 protein; NR, not yet recruiting*.

### Published Clinical TCR-T Cell Therapies in Hematological Malignancies

The clinical application of gene-modified T cell therapy has led to unprecedented response rates in leukemia and lymphoma patients treated with anti-CD19 CAR-T cells. Although CAR-T cell therapy has shown significant efficacy in CD19+ hematological cancers such as lymphoma and leukemia, life-threatening side effects such as CRS and neurotoxicity limit the clinical application of CAR-T cell therapy. Eureka Therapeutics has published preclinical data on its proprietary antibody-TCR (AbTCR) platform, proving that AbTCR-T cells have the same anticancer effects as existing anti-CD19 CAR-T cells in a mouse model (102); however, the production of inflammatory cytokines is significantly reduced in AbTCR-T cells, reducing the risk of CRS. Safer T cell therapy can provide patients with a larger therapeutic window while reducing the direct and indirect costs for patients and the entire health care system (Table 8).

**Table 8:** Published clinical TCR-T therapy for hematological malignancies.

**Target**	**Disease**	**Vector**	**Pretreatment**	**Dose**	**No. of patients**	**Phage**	**Response**	**Country**	**Reference**
WT1	Acute myeloid leukemia; MDS	Retrovirus	Cytosine arabinoside and aclarubicin	2 × 10^8^−5 × 10^9^/person	8	I	50% remission	Japan	(106)

### Published Clinical TCR-T Cell Therapies in Solid Tumors

Researchers isolated endogenous TCRs, engineered them, and introduced these modified T cells into the human body. In this way, the number of T cells with the ability to target cancer cells will increase, and this is expected to help identify and target multiple solid tumors and hematological tumors (Table 9).

**Table 9:** Published clinical TCR-T therapy for solid tumors.

**Target**	**Disease**	**Vector**	**Pretreatment**	**Dose**	**No. of patients**	**Phage**	**Response**	**Country**	**References**
MART-1	Metastatic melanoma	Retrovirus	Chemotherapy	1.5 × 10^9^−1.07 × 10^11^ /person	20	II	30% objective antitumor response	United States	(107)
Gp100	Metastatic melanoma	Retrovirus	Chemotherapy	1.8 × 10^9^−1.1 × 10^11^ /person	16	II	19% objective antitumor response	United States	(107)
CEA	Metastatic colorectal	Retrovirus	Chemotherapy	2–4 × 10^8^/person	3	–	Grade 2/3 diarrhea	United States	(108)
NY-ESO-1	Metastatic melanoma/synovial cell sarcoma	Retrovirus	Chemotherapy	1.6 × 10^10^−1.3 × 10^11^/person	17	I	2 complete remission; 1 partial remission	United States	(109)
NY-ESO-1	Multiple myeloma	Lentivirus	Chemotherapy	Average dose of 2.4 × 10^9^/person	20	I/II	80% remission;	United States	(104)
MAGE-A3	Metastatic melanoma/multiple myeloma	Lentivirus	CY	2.4–5.3 × 10^9^/person	2	III/IV	2 died for cardiac toxicity	United States	(110)
MAGE-A4	Esophageal cancer	Retrovirus	Surgery; radiotherapy; chemotherapy	2 × 10^8^−5 × 10^9^/person	10	I	7/10 tumor progression	Japan	(111)

### Differences Between CAR-T Cell and TCR-T Cell Therapies

CARs can recognize not only peptide antigens but also carbohydrate and glycolipid antigens, which enlarges the pool of targetable cancer antigens. CAR-T cell therapy is not restricted by MHC class. CAR-T cells can target nonprotein glycolipid antigens of cancer cells to recognize antigens in multiple dimensions. The problem with the treatment of solid and hematological tumors by CAR-T cells is that the target antigens are all cell surface proteins. Even if CAR-T cells have a potent killing effect on tumor cells, without appropriate, specific cell surface target proteins, CAR-T cell therapy cannot have a real impact. Because MHC molecules can present peptide chains obtained from the cell surface or intracellular proteins, TCRs can target more antigens than CARs. These antigens, including CEA, HER-2, CD19, gp100, MART-1, MAGA-A3, and NY-ESO-1, are suitable for TCR-T cell therapy. TCRs target intracellular proteins with tumor specificity. These antigens include neo-antigens generated by random mutations in tumor DNA and so-called cancer testis antigens (82). These antigens are usually expressed in only some tumors and genital tissues, while MHC molecules are not expressed in genital tissues. TILs and TCRs can recognize only the antigens presented by specific MHC molecules and may escape immune surveillance due to the downregulation or mutation of MHC molecules in tumor cells, resulting in clinical limitations (25). TCR-based therapies may be superior to CAR-based therapies because TCR-T cells use natural T cell signaling mechanisms (112).

The dosage of CAR-T cell therapy is two to three orders of magnitude lower than that needed with TILs and TCR-T cells. Because CAR-T cell therapies have a clear target and a high specificity for recognizing tumor surface antigen and are able to overcome MHC restriction, the number of single transfusion cells needed for CAR-T cell therapy is far smaller than that needed for TCR-T cell therapy or TIL therapy to generate the same therapeutic effect.

## Discussion

Immunotherapy, unlike traditional therapies, directly acts on cancer cells by recruiting the autoimmune system and using it to attack tumors. The anti-tumor immune response can continuously recognize and memorize tumor antigens and increasingly expand with time; this cycle can be repeated to strengthen the anti-tumor immune response. With expansion of the immune response, some cytotoxic T cells differentiate into mature memory T cells, which can provide long-term immune memory even when primary antigen stimulation does not exist. Therefore, immunotherapy can bring lasting benefits and long-term survival to patients.

The development of a new drug or therapy is not easy, and it often costs billions of dollars. Juno Therapeutics is committed to the research of tumor cell immunity and is one of the pioneers in this field. At present, research on CAR-T cell products targeting CD19 is relatively extensive, and the two approved products target CD19 for the treatment of hematological tumors. Globally, the R&D pipeline of CAR-T cells has expanded rapidly, including the exploration of new targets, such as BCMA, CD123, and CD33, as well as the expansion to new indications, such as the progression of hematological tumors to solid tumors. A number of companies around the world have been pushing their projects to the clinical stage, and it is expected that there will be CAR-T cell products for different tumors in the future. With the promotion of new structures in clinical trials and improvements in production technology, the third and fourth generations of CAR-T cell products are expected to have better efficacy. TCR-T therapies targeting solid tumor targets such as WT-1, L1CAM, ROR-1, MUC-16, LewisY, HPV-16, etc. are also undergoing clinical trials. For solid tumors, TCR-T therapies may be more advantageous. Many companies around the world such as Kite Pharma, JunoTherapeutics, Adaptimmune Therapeutics, etc. are all committed to the development of TCR-T immunotherapy, hoping to bring feasible treatment plans.

The preparation of engineered T cells still depends on traditional manual operation, such that cell quality and stability are difficult to guarantee. Moreover, the curative effect is greatly reduced. With the continuous improvement of engineered T cell technologies, it is foreseeable that automatic production of engineered T cells will be a major trend in the future. We also look forward to engineered T cell therapy becoming more technically mature, safer, and efficient. Tumor immunotherapy will bring hope to many cancer patients soon. Some of the greatest challenges in developing cell therapies include the lack of preclinical models to assess the safety and efficacy of these complex therapies and the responses to safety issues identified in early clinical studies. In addition, there are still many obstacles in the treatment of solid tumors.

It is expected that, due to technological advances in engineering T cells, gene editing, and cell manufacturing, the foundations of T cell-based therapies will be extended to other cell types in the future, such as induced pluripotent stem cells, hematopoietic stem cells, and NK cells. The indications will also transcend cancer and are expected to include infectious diseases, organ transplantation, and autoimmune diseases.

## Author Contributions

LZ drafted the manuscript, during which she received carefully guidance and support from YC. All authors read and approved the final manuscript.

### Conflict of Interest

The authors declare that the research was conducted in the absence of any commercial or financial relationships that could be construed as a potential conflict of interest.
